# The Potential Role of Polymethyl Methacrylate as a New Packaging Material for the Implantable Medical Device in the Bladder

**DOI:** 10.1155/2015/852456

**Published:** 2015-02-01

**Authors:** Su Jin Kim, Bumkyoo Choi, Kang Sup Kim, Woong Jin Bae, Sung Hoo Hong, Ji Youl Lee, Tae-Kon Hwang, Sae Woong Kim

**Affiliations:** ^1^Department of Urology, Seoul St. Mary's Hospital, 222 Banpo-daero, Seocho-gu, Seoul 137701, Republic of Korea; ^2^Department of Mechanical Engineering, Sogang University, Seoul, Republic of Korea

## Abstract

Polydimethylsiloxane (PDMS) is used in implantable medical devices; however, PDMS is not a completely biocompatible material for electronic medical devices in the bladder. To identify novel biocompatible materials for intravesical implanted medical devices, we evaluated the biocompatibility of polymethyl methacrylate (PMMA) by analyzing changes in the levels of macrophages, macrophage migratory inhibitory factor (MIF), and inflammatory cytokines in the bladder. A ball-shaped metal coated with PMMA or PDMS was implanted into the bladders of rats, and after intravesical implantation, the inflammatory changes induced by the foreign body reaction were evaluated. In the early period after implantation, increased macrophage activity and MIF in the urothelium of the bladder were observed. However, significantly decreased macrophage activity and MIF in the bladder were observed after implantation with PMMA- or PDMS-coated metal in the later period. In addition, significantly decreased inflammatory cytokines such as IL-1*β*, IL-6, and TNF-*α* were observed with time. Based on these results, we suggest that MIF plays a role in the foreign body reaction and in the biocompatible packaging with PMMA for the implanted medical devices in the bladder.

## 1. Introduction

Numerous researchers have studied the development and application of implantable medical devices, and devices such as pacemakers and cardiac defibrillators are widely used [1]. In the field of urology, several implantable medical devices are used such as urethral or double-J catheters and InterStim (Medtronic, Minneapolis, MN, USA), an implantable device that treats overactive bladder by stimulating the sacral nerve [2]. Recently, several studies have developed implantable sensors to monitor intravesical pressure or volume changes [3–5]. Implantable devices that monitor real-time changes in intravesical pressure or volume are necessary for patients suffering from neurogenic voiding dysfunction because these devices prevent renal damage induced by abnormally increased intravesical pressure [6]. Moreover, the characteristics of voiding dysfunction are very diverse and depend on the patient condition; therefore, implantable intravesical devices capable of real-time monitoring are necessary to satisfy patient need [7]. To this end, we developed implantable bladder pressure- and volume-monitoring sensors [8, 9].

In the clinical application of implantable medical devices, both function and biocompatibility are important. Packaging with biocompatible polymers provides biocompatibility and maintains the function of implantable bioelectronics [1]. Polydimethylsiloxane (PDMS) is a biocompatible polymer used in urologic medical devices such as urethral catheters and can be used for the coating of implantable electronic sensors in the bladder. Although PDMS has beneficial biocompatibility in the bladder, identifying new biocompatible materials with higher impact resistance and lower electronic fluctuation for the implanted electronic sensors in the bladder is necessary. Polymethyl methacrylate (PMMA) is widely used for the construction of medical devices such as microsensors, drug delivery applications, bone cement, and denture base to hold teeth during mastication [1, 10, 11]. Specially, PMMA is used as bone cement and a denture base because it demonstrates high scratch and impact resistance. In addition, a recent study demonstrated that PMMA-coating reduced charge fluctuations in metal oxide nanowires, and PMMA-coating stabilized the electrical characteristics [12]. Therefore, PMMA may be a new biocompatible coating material that possesses better characteristics compared with PDMS for use in electronic sensors, which move freely in the bladder. However, studies regarding the biocompatibility of PMMA in the bladder remain lacking [1, 11, 13, 14]; therefore, we evaluated the inflammatory responses to PMMA and compared the responses induced by PDMS, which is already regarded as biocompatible in the bladder.

After implantation of a foreign material, the body reaction occurs as an inflammatory response, and macrophages and inflammatory cytokines play important roles in this response. Moreover, the roles of macrophages in various tissues and changes in these cells in response to biomaterials have been well established.

The cytokine, macrophage migration inhibitory factor (MIF), is involved in the inflammatory response and is known to regulate the inflammatory response in various inflammatory diseases such as rheumatoid arthritis, pulmonary inflammation, and sepsis [15–17]. Many investigators have also noted the presence of MIF in the urothelium and the role of MIF in cystitis; therefore, MIF appears to be related to bladder inflammation [18]. After foreign materials are implanted in the bladder, they directly contact the urothelium. The urothelium has an important role in the first line of bladder defense in response to pathogens and it influences the response to foreign materials. For these reasons, studying MIF changes in the bladder is necessary because MIF abundantly exists in the urothelium and affects bladder inflammation. However, a shortage of information exists regarding MIF changes after foreign material implantation, and a few researchers have reported changes in MIF during foreign body reaction [19].

Therefore, in this study, we evaluated changes in macrophages and inflammatory cytokines after the intravesical implantation of PMMA to investigate its biocompatibility in the bladder. We also investigated MIF changes and the role of MIF in the body reaction to foreign biomaterials.

## 2. Materials and Methods

### 2.1. Animals

White male Sprague-Dawley (SD) rats aged 8 weeks with weight distribution ranging from 250 to 300 g (*n* = 120) were used in this study. The rats were divided into the following 4 groups: the control group (*n* = 30), the sham-operated group (*n* = 30), PDMS-coating (PDMS-coated metal group; *n* = 30), and PMMA-coating (PMMA-coated metal group; *n* = 30). The experimental protocol was approved by the Catholic University Animal Ethics Committee (CUMC-2014-0013-01).

### 2.2. Coating with PDMS and PMMA

The 2 mm sized ball-shaped metal piece was immersed in PDMS solution (Sylgard 184; Dow Corning, Seoul, Korea; silicone elastomer : curing agent Z, 10 : 1) and heated at 80°C for 2 hours. The 2 mm sized ball-shaped metal piece was immersed in the solution of PMMA (Aldrich, St. Louis, MO, USA), dissolved in 2-ethoxyethyl acetate(2-EEA) at 80°C, and then dried for 2 hours at 180°C in a dry oven.

### 2.3. Surgical Procedures

Tiletamine (Zoletil) 0.2 mL was injected intraperitoneally to anesthetize the animals. A lower midline incision was made and the bladder was exposed and incised. In the sham-operated group (*n* = 30), no further surgical manipulation was made. The PDMS- and PMMA-coated metals were placed in each bladder. Next, the bladder was closed with absorbable 4/0 polydioxanone, and the abdomen was closed with 3/0 chromed catgut and silk.

### 2.4. Bladder Histological Evaluation

To evaluate chronic bladder inflammatory change, rats (*n* = 40; 10 control, 10 sham-operated, 10 implanted with PDMS-coated metal and 10 implanted with PMMA-coated metal) were sacrificed after 4 weeks. The bladders were collected and fixed in 4% neutral paraformaldehyde for 1 day. For the preparation of the fixed tissues for light microscopy, the tissues were dehydrated with alcohol, embedded in paraffin, sectioned in 5-*μ*m sections with a microtome, and stained with hematoxylin and eosin (H&E). Histological images of the bladder were obtained using a light microscope at 100x magnification. In each section, at least 5 fields were selected at random. Bladder inflammation was assessed by a pathologist in blinded fashion using the following four-point scoring system: 0, morphologically unremarkable with no or minimal inflammation or epithelial changes; (1) mild inflammatory infiltrate within the lamina propria with scattered lymphocytes or monocytes, accompanied by mild chronic edema, hemorrhage, or urothelial changes; (2) moderate inflammatory infiltrate in the lamina propria and focal extension of the inflammation into the muscularis propria, accompanied by moderate chronic edema, hemorrhage, fibrin deposition, or urothelial changes; (3) severe inflammation in the lamina propria and muscularis propria associated with other significant findings, such as urothelial ulceration, severe chronic edema, hemorrhage, and fibrin deposition [20].

### 2.5. Immunofluorescence Staining to Visualize Macrophages and MIF

The rats were sacrificed after 1, 2, and 4 weeks, and the sectioned bladders were deparaffinized, rehydrated, treated with 3% hydrogen peroxide to block endogenous peroxidase, rinsed, and then kept in 0.01 M PBS. Next these sectioned bladders were microwaved to retrieve the antigen and then exposed to a 10% normal serum to block any nonspecific reactions. Then, the sections were incubated at 4°C overnight with anti-MIF antibody (diluted 1 : 200; Abcam, Cambridge, UK) and anti-macro antibody (diluted 1 : 200; Abcam, Cambridge, UK) for MIF/Macro costaining. After washing with PBTx, the samples were then incubated with secondary antibody [Alexa Fluor 568 goat anti-rabbit IgG; Alexa Fluor 488 goat anti-mouse IgG Invitrogen, Carlsbad, CA, USA] in dilute solution at room temperature for 1 h. After washing with PBTx, a coverslip was mounted on the slide using mounting medium with 4,6-diamino-2-phenyl-indole (DAPI; Vector Labs Burlingame, CA, USA) to observe the cell nuclei. Digital images were obtained using an Olympus BX51 fluorescence microscope.

### 2.6. Macrophage, MIF, and Cytokine Analyses

The rats (*n* = 120) were sacrificed after 1, 2, and 4 weeks, and the bladder samples were collected and frozen at −80°C. The concentrations of IL-1*β* (Invitrogen, San Diego, CA, USA) IL-6, and TNF-*α* (R & D Systems, Minneapolis, MN, USA) were measured by enzyme-linked immunosorbent assay (ELISA) according to manufacturer's instructions. Macrophage (USCN life Science, Inc., Wuhan, China) and MIF (CUSABIO Biotech, Wuhan, China) concentrations were measured by enzyme-linked immunosorbent assay (ELISA) according to manufacturer's instructions. IL-1*β*, TNF-*α*, MIF, and macrophages were measured using a spectrophotometer at 450 nm and IL-6 was measured using a spectrometer at 570 nm.

### 2.7. Statistical Analysis

The statistical analysis was performed using SPSS 15.0 (SPSS Inc., Chicago, IL, USA). The data were expressed as the means ± standard deviations. The data for each group were compared using a one-way ANOVA and Bonferroni post hoc test. The significance was set at *P* < 0.05.

## 3. Results

### 3.1. Comparison of Chronic Bladder Inflammatory Changes

**Figure 1:**
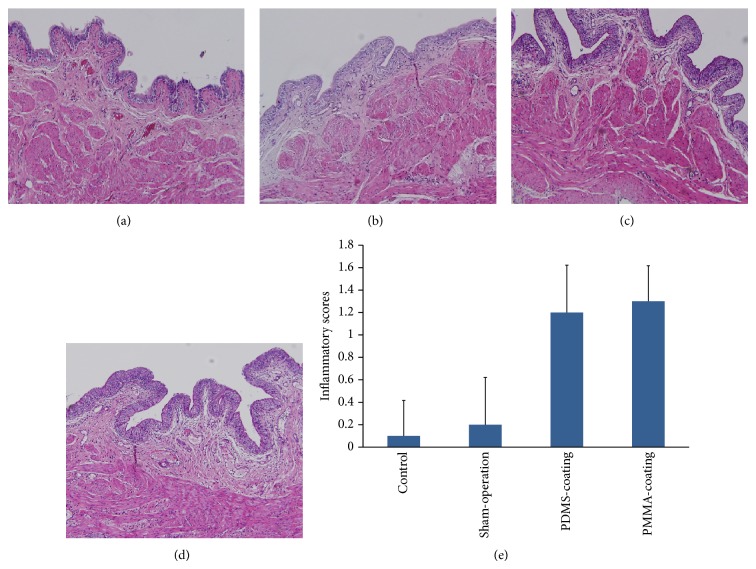
Inflammatory changes of the bladder at 4 weeks after intravesial implantation; the control (a) and sham-operation (b), in rats implanted with PDMS- (c) and PMMA- (d) coating. Mild degree of inflammation was observed in the rats implanted with PDMS- and PMMA-coated metal (e). Magnification: ×100.

**Figure 2:**
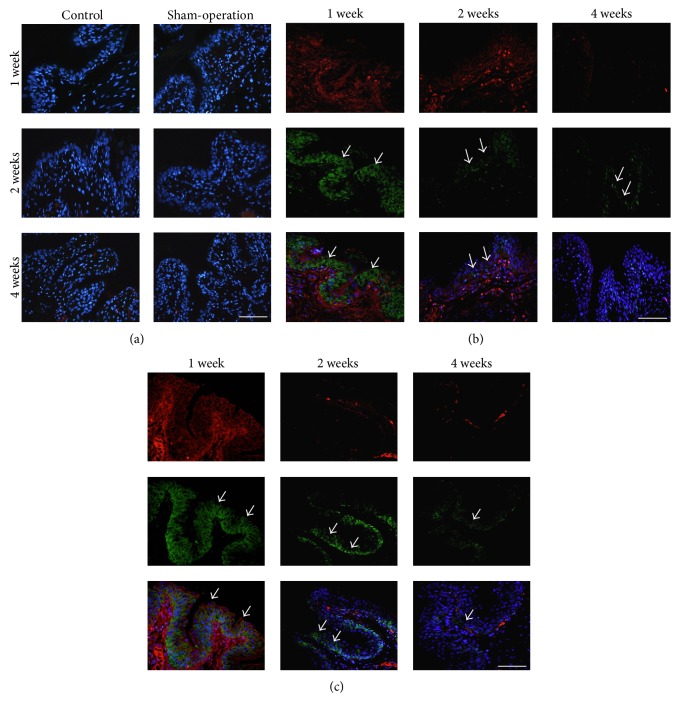
Macrophages and MIF expression in the urothelium from the rats at 1, 2, and 4 weeks after implantation. The control, sham-operated (a), PDMS- (b), and PMMA- (c) coated metal were shown. The figure shows macrophages immunostaining (red immunofluorescence), MIF immunostaining (green immunofluorescence), and a DAPI nuclear stain (blue immunofluorescence). Macrophages and MIF were not expressed in the control and sham-operated groups (a) at 1, 2, and 4 weeks. Increased expression of macrophages and MIF was observed in the urothelium after PDMS- and PMMA-coated metal implantation at 1 week (b and c). MIF was detected in basal and intermediate layers of the urothelium at 1 week. Macrophages and MIF expression were decreased and only detected in basal layer at 2 weeks and rarely expressed at 4 weeks in the rats implanted with PDMS- and PMMA-coated metal (b and c; arrows). Scale bar = 100 *μ*m.

In rats implanted with PDMS- and PMMA-coated metal, mild inflammatory changes were observed compared with the control and sham-operated group (Figure 2(a)). The degree of bladder inflammation in the rats implanted with PDMS- and PMMA-coated metal was mild at 4 weeks (Figure 1(b)). Moreover, the degree of inflammation in the rats implanted with PMMA-coated metal was similar to the rats implanted with PDMS-coated metal at 4 weeks.

### 3.2. Macrophage and MIF Expression in the Urothelium at 1, 2, and 4 Weeks after Implantation

At 1 week after implantation with PDMS- and PMMA-coated metal, increased macrophage and MIF expression were observed in the bladder urothelium (Figures 2(b) and 2(c)). This increase in expression is regarded as the early inflammatory reaction to the foreign material. Decreased expression of macrophages and MIF was observed in the rats implanted with PDMS- and PMMA-coated metal at 2 weeks. The macrophages and MIF were rarely expressed in the rats implanted with PDMS- and PMMA-coated metal at 4 weeks.

### 3.3. The Changes of Macrophages and MIF at 1, 2, and 4 Weeks after Implantation

After implantation, significantly increased macrophage and MIF activity in the bladder were observed in the rats implanted with PDMS- and PMMA-coated metal compared with the control and sham-operated groups at 1 week (Table 1). At 2 weeks, decreased macrophage and MIF activity in the bladder were observed in the rats implanted with PDMS- and PMMA-coated metal. At 4 weeks, macrophage and MIF activity in the bladder were significantly decreased in the rats implanted with PDMS- and PMMA-coated metal, and the differences between the control, sham-operated groups, and the rats implanted with PDMS- and PMMA-coated metal were not significant.

### 3.4. The Changes in the Inflammatory Cytokine Levels at 1, 2, and 4 Weeks after Implantation

After implantation with PDMS- and PMMA-coated metal, significantly increased IL-1*β*, IL-6, and TNF-*α* bladder levels were observed compared with the control and sham-operated groups at 1 week (Table 2). Although no significant differences were observed between the rats implanted with PDMS- and PMMA-coating, the increase in IL-1*β*, IL-6, and TNF-*α* levels after implantation with PMMA-coated metal was lower compared with that in the rats implanted with PDMS-coated metal at 1 week. Decreased IL-1*β*, IL-6, and TNF-*α* levels were noted in the rats implanted with PDMS- and PMMA-coated metal at 2 weeks. At 4 weeks, IL-1*β*, IL-6, and TNF-*α* were higher in the rats implanted with PDMS- and PMMA-coated metal compared with the control and sham-operated groups; however, no significant difference was observed.

## 4. Discussion

In this study, we observed significantly decreased macrophage activity and lower levels of inflammatory cytokines that are associated with the foreign body reaction in the bladders of rats after PMMA-coating, and this result indicates that biocompatibility of PMMA is similar to PDMS, which is used as the material of urethral catheters in the bladder. Consistent with these observed changes, MIF expression was significantly decreased in the urothelium of rats implanted with PDMS- or PMMA-coated metal. In particular, MIF changes in the bladder need to be examined, because MIF abundantly exists in the urothelium, which is the first gate that meets foreign materials in the bladder. In addition, the present study of changes in MIF after the implantation of PDMS- and PMMA-coated metal can be considered the first investigation of this subject in the bladder. Although MIF is known to play a role in various inflammatory conditions, few studies have investigated changes in MIF activity and the role of MIF in the foreign body reaction to biomaterials implanted in human tissue [15–17].

PDMS and PMMA are widely used biomaterials and are used in several implanted medical devices that have already been developed or that are in development [11, 13, 14, 21]. Of these polymers, PDMS is used in urethral catheters, which help drain urine from the bladder. Urethral catheters constructed of PDMS are used for patients who need indwelling urethral catheters for long periods because these catheters induce fewer urethral catheter-associated problems such as UTIs and encrustation compared with latex catheters [21, 22]. In addition, we previously reported biocompatibility of PDMS in the bladder wall [23]. Although PDMS is currently the most reliable biomaterial for intravesical implanted medical devices, PDMS also possesses several limitations such as relatively low impact resistance. In addition, we sought better coating materials that do not affect electronic function of the sensors in the bladder. Most previous studies on the application and biocompatibility of PMMA devices have investigated orthopedic implants due to their good impact resistance; however, a recent study used PMMA as a coating material for latex gloves and reported that coatings with PMMA-chitosan nanoparticles reduced the latex cytotoxicity [24]. For these reasons, we selected PMMA as a candidate material for coating intravesically implanted medical devices. The degree of impact resistance of PMMA depends on the curing environment and we followed the recommended protocol to show higher surface hardness in this study [25].

Macrophages are markers that are known to be associated with the foreign body reaction; therefore, these cells are widely used as indicators to evaluate the biocompatibility of implanted medical devices [26]. As with other biomaterials, decreased macrophage activity was observed in the bladders of the rats implanted with PDMS- or PMMA-coated metal at 4 weeks. Additionally, as the macrophage activity decreased, lower levels of the IL-1*β*, IL-6, and TNF-*α* cytokines were noted in the bladders of the rats implanted with PDMS- or PMMA-coated metal. Lower inflammatory changes in the rat bladders implanted with PMMA-coated metal similar to the control and sham-operated animals support this hypothesis. In addition, inflammatory cytokines such as IL-1*β*, IL-6, and TNF-*α* after PMMA-coating were less increased compared with PDMS-coating at 1 week after intravesical implantation, although the difference was not significant. These results suggest that the mild early inflammatory reaction to the foreign materials occurs after using PMMA to coat implanted medical devices in the bladder. Thus, for the first time, we observed that PMMA is a biocompatible coating material that can be used in the intravesical environment. Moreover, PMMA may be a biocompatible material with similar low inflammatory responses with PDMS, which is already known to be safe in the bladder.

MIF is a proinflammatory cytokine and the up- or downregulation of MIF is associated with various inflammatory diseases. Moreover, several recent studies reported the presence of MIF in the urothelium of the bladder, and increased MIF expression in the urothelium is also associated with bladder inflammation [18, 27, 28]. In addition, several studies have reported changes in MIF during inflammatory conditions that are related to the foreign body reaction to implanted medical devices [19, 29–31]. Therefore, we assumed that MIF in the urothelium may play a role in bladder inflammation induced by contact between the urothelium and implanted medical devices. Consistent with our hypothesis, changes in MIF were observed in the urothelium after intravesical foreign body implantation in this study. MIF expression was significantly higher in the rats that had been intravesically implanted compared with the rats implanted with PDMS- or PMMA-coated metal at an early period after implantation. Moreover, in each animal that was intravesically implanted with PDMS- or PMMA-coated metal, the degree of MIF expression and levels decreased as the macrophage level reduced in the bladder over time. These results are consistent with the findings of previous researchers showing that increased MIF levels are correlated with the severity of the foreign body reaction [19, 29–31]. If an implanted material is not biocompatible, macrophages that are recruited to the implant site release inflammatory cytokines such as IL-1*β*, IL-6, and TNF-*α*, which induce chronic inflammation. In addition, we assumed that MIF plays a role in the production of IL-1*β*, IL-6, and TNF-*α* after the implantation of foreign materials in the bladder, because prior investigators have reported that MIF affects the release of IL-1*β*, IL-6, and TNF-*α* in other inflammatory diseases [32, 33]. Cox et al. [32] showed that the presence of MIF induces autoimmune neuroinflammation by modulating IL-1*β*, IL-6, TNF-*α*, and macrophage accumulation. Other researchers have also found that the use of an MIF inhibitor decreased IL-6-mediated inflammation in nasal polyps. Therefore, we hypothesized that not only macrophages but also MIF may increase IL-1*β*, IL-6, and TNF-*α* release in the bladder. For this reason, the observed early increase in inflammatory cytokine release after intravesical implantation may be due to the cumulative effect of macrophages and MIF in the urothelium. Furthermore, the significantly decreased release of inflammatory cytokines that is regulated by macrophages and MIF with time indicates that PMMA- and PDMS-coatings are biocompatible in the bladder.

Although the present study demonstrated the biocompatibility of PMMA in the bladder and the role of MIF in the urothelium in response to foreign body reactions, several limitations exist. In this study, we performed ELISA analysis of macrophages and MIF in the whole bladder tissue to show the general molecular changes of the bladder. The entire bladder was changed according to the foreign body reactions although the urothelium is the first gate to meet the foreign materials in the bladder. However, analysis of macrophages and MIF changes in each part of the urothelium and detrusor muscle appears to be necessary for the better understanding of exact molecular changes in the bladder, and we evaluated the biocompatibility in the rats. Rats tend not to have strong foreign body reactions compared with humans; therefore, the results obtained using rats may not be appropriate despite the importance of using rats in preclinical studies. Therefore, to improve the value of these results, further investigations are necessary, including studies using real sensors coated with PMMA.

## 5. Conclusions

In this study, PMMA-coating reduced MIF expression and macrophage activity in the bladder, and these changes decreased the production of inflammatory cytokines that exacerbate the foreign body reaction. Moreover, the present findings demonstrate that MIF may participate in the foreign body reaction in the urothelium of the bladder and that changes in MIF expression are influenced by the characteristics of intravesically implanted materials. Therefore, we suggest that PMMA may be a useful candidate biomaterial for the packaging of medical devices for implantation in the bladder and that MIF affects the inflammation associated with the foreign body reaction in the bladder.

## Figures and Tables

**Table 1:** The changes of macrophages and MIF in the bladder.

	1 week	2 weeks	4 weeks
Control			
Macrophages (pg/mL)	1.0 ± 0.07	0.9 ± 0.08	0.9 ± 0.08
MIF (pg/mL)	211.0 ± 0.06	269.5 ± 0.01	308 ± 0.02
Sham-operation			
Macrophages (pg/mL)	1.3 ± 0.08	1.0 ± 0.1	1.2 ± 0.06
MIF (pg/mL)	209.0 ± 0.07	298.0 ± 0.03	305.3 ± 0.02
PDMS-coating			
Macrophages (pg/mL)	5.5 ± 0.11^∗,∗∗^	3.6 ± 0.05	1.5 ± 0.05
MIF (pg/mL)	878.3 ± 0.18^∗,∗∗^	599.7 ± 0.13	458.3 ± 0.08
PMMA-coating			
Macrophages (pg/mL)	5.2 ± 0.13^∗,∗∗^	2.8 ± 0.05	1.8 ± 0.01
MIF (pg/mL)	855.4 ± 0.13^∗,∗∗^	605.5 ± 0.24	540 ± 0.05

^*^ *P* < 0.05 compared with the control,  ^**^ *P* < 0.05 compared with sham-operation.

**Table 2:** The changes of inflammatory cytokines, IL-1*β*, IL-6, and TNF-*α*, in the bladder.

	1 week	2 weeks	4 weeks
Control			
IL-1*β* (pg/mL)	85.7 ± 0.02	83.5 ± 0.01	84.9 ± 0.01
IL-6 (pg/mL)	253.8 ± 0.05	322.1 ± 0.01	302.9 ± 0.01
TNF-*α* (pg/mL)	42.0 ± 0.01	38.4 ± 0.01	37.4 ± 0.02
Sham-operation			
IL-1*β* (pg/mL)	84.0 ± 0.02	85.3 ± 0.03	86.0 ± 0.01
IL-6 (pg/mL)	249.3 ± 0.03	308.9 ± 0.01	311.7 ± 0.05
TNF-*α* (pg/mL)	39.6 ± 0.02	40.1 ± 0.03	38.7 ± 0.01
PDMS-coating			
IL-1*β* (pg/mL)	320.0 ± 0.05^∗,∗∗^	240.4 ± 0.01	148.7 ± 0.02
IL-6 (pg/mL)	602.4 ± 0.05^∗,∗∗^	515.7 ± 0.02	388.2 ± 0.03
TNF-*α* (pg/mL)	101.3 ± 0.01^∗,∗∗^	70.5 ± 0.01	47.2 ± 0.02
PMMA-coating			
IL-1*β* (pg/mL)	255.7 ± 0.05^∗,∗∗^	189.7 ± 0.04	150.1 ± 0.01
IL-6 (pg/mL)	456.3 ± 0.03^∗,∗∗^	548.2 ± 0.01	389.4 ± 0.04
TNF-*α* (pg/mL)	87.6 ± 0.02^∗,∗∗^	62.8 ± 0.23	48.5 ± 0.09

^*^ *P* < 0.05 compared with the control,  ^**^ *P* < 0.05 compared with sham-operation.
